# Development of a NS2B/NS3 protease inhibition assay using AlphaScreen^®^ beads for screening of anti-dengue activities

**DOI:** 10.1016/j.heliyon.2018.e01023

**Published:** 2018-12-08

**Authors:** Muhammad Asyraf Abduraman, Maywan Hariono, Rohana Yusof, Noorsaadah Abd Rahman, Habibah A. Wahab, Mei Lan Tan

**Affiliations:** a Malaysian Institute of Pharmaceuticals & Nutraceuticals, National Institutes of Biotechnology Malaysia (NIBM), Ministry of Science, Technology and Innovation Malaysia, Pulau Pinang, Malaysia; b Pharmaceuticals Drug Simulation Laboratory (PhDS), School of Pharmaceutical Sciences, Universiti Sains Malaysia, Pulau Pinang, Malaysia; c Department of Molecular Medicine, Faculty of Medicine, University of Malaya, Kuala Lumpur, Malaysia; d Department of Chemistry, Faculty of Science, University of Malaya, Kuala Lumpur, Malaysia; e Advanced Medical & Dental Institute, Universiti Sains Malaysia, Pulau Pinang, Malaysia

**Keywords:** Bioinformatics, Biotechnology

## Abstract

**Background:**

Dengue infection is an endemic infectious disease and it can lead to dengue fever, dengue hemorrhagic fever, and/or dengue shock syndromes. Dengue NS2B/NS3 protease complex is essential for viral replication and is a primary target for anti-dengue drug development. In this study, a NS2B/NS3 protease inhibition assay was developed using AlphaScreen^®^ beads and was used to screen compounds for their protease inhibition activities.

**Methods:**

The assay system utilized a known NS2B/NS3 peptide substrate, a recombinant of NS2B/NS3 protease with proprietary StrepTactin^®^ donor and nickel chelate acceptor beads in 384-well format.

**Results:**

The optimized assay to screen for NS2B/NS3 protease inhibitors was demonstrated to be potentially useful with reasonable zʹ factor, coefficient variance and signal to background ratio. However, screening of synthesized thioguanine derivatives using the optimized AlphaScreen^®^ assay revealed weak NS2B/NS3 inhibition activities.

**Conclusion:**

The AlphaScreen^®^ assay to screen for NS2B/NS3 protease inhibitors is potentially applicable for high throughput screening.

## 1 Introduction

Dengue infection is an endemic infectious disease found mainly in tropical and subtropical regions. According to the World Health Organization (WHO), nearly 50–100 million dengue infection cases occur every year. An estimated half a million people with severe dengue infection require hospitalization each year, a large proportion of whom are children and about 2.5% of those affected with dengue, succumbed to this infection [1]. Dengue fever is caused by a vector-borne virus transmitted by *Aedes aegypti* mosquitoes. Dengue virus (DENV) is a member of the flavivirus genus within the family of *Flaviviridae* [2, 3]. DENV possesses four distinct serotypes that are closely related, however infection with one serotype does not provide complete protection against the other three serotypes [2]. Secondary infection with a heterologous serotype is a risk factor for developing dengue hemorrhagic fever or dengue shock syndrome [4].

DENV is categorized as an enveloped virus containing a 10.7kb single-stranded RNA genome of positive strand polarity [5, 6, 7]. The RNA genome is transcribed into a single polyprotein containing 3 structural proteins [envelope (E), pre-membrane (prM) and capsid (C)] and 7 non-structural proteins (NS1, NS2A, NS2B, NS3, NS4A, NS4B and NS5) [8, 9, 10]. Co- and post-translational cleavage are aided by a variety of host and viral proteases resulting in distinct structural proteins [7]. Proteolytic processing in the region of the structural proteins is mediated by a host cell signal peptidase that is located within the endoplasmic reticulum [11]. Cleavages at the NS2A/NS2B, NS2B/NS3, NS3/NS4A, and NS4B/NS5 sites are catalyzed by the virus encoded two-component protease NS2B/NS3. This protease also cleaves C/prM and various internal sites in C, NS2A, NS3 and NS4A [12]. The cleavage activity of NS3 is aided by NS2B protein as a cofactor [13]. The NS2B/NS3 protease complex is essential for viral replication and is a primary target for the development of anti-dengue drugs [9, 14, 15].

The active site of the NS3 serine protease carries the catalytic triad, comprising of three amino acid residues, namely H51, D75 and S135. The NS2B act as a cofactor of NS3 serine protease for optimal catalytic activity [8]. Formation of hydrogen bond between H51 and D75, allows H51 to deprotonate serine causing S135 to play a role in fragmenting substrate [16]. In addition to the catalytic triad, the active site of NS3 also contained 4 sub-pockets, namely sub-pocket 1 (D129, S135, Y150, Y161), sub-pocket 2 (D75, D82, G83, N84, N152), sub-pocket 3 (F85, Q86, L87) and sub-pocket 4 (V154). Interactions with sub-pocket residues alongside with catalytic triad aid in the binding affinity and contribute to the inhibitory characteristics of the compounds [6, 17, 18].

Currently, various efforts are underway to find strategies to manage this infectious disease, and this includes screening for new chemical entities targeted against structural and/or nonstructural proteins involved in the viral replication and the development of dengue vaccine. The first licensed dengue vaccine, Dengvaxia^®^ has been evaluated in 2 parallel Phase 3 randomized clinical trials, in Asia and Latin America. However, vaccine efficacy ranged from 31.3% to 79%, reflecting at least in part the variability of baseline seropositivity and circulating serotypes, both of which affect the performance of the vaccine [19, 20]. At present, there is no antiviral therapy that is available for the prevention and treatment of acute dengue virus infection. However, efforts were still ongoing, for example, the cyclohexenyl chalcone derivatives of a Zingiberaceae species, *Boesenbergia Rotunda* (L.) were reported to inhibit DENV2 protease [6, 21]. Panduratin A and 4-hydroxypanduratin A, compounds isolated from this plant were found to exhibit modest inhibitory activities, specifically 65–80% inhibition at 80 ppm (200 μM) [21]. In addition to natural products, repository libraries such as National Cancer Institute, USA (NCI) library which maintains a large number of compounds from both synthetic and natural resources is a valuable repository to source for potential anti-dengue compounds. In our preliminary unpublished work, screening of NCI compounds (diversity sets) for anti-dengue properties was carried out using the protease assay as described by Yusof and co-workers [22]. In this study, further efforts were undertaken to synthesize compounds based on structure-activity relationship modelling.

One of the important reasons contributing to the lack of antiviral drugs to treat dengue would be the challenging nature of identifying chemical compounds with specific activities. Previously, cytopathic and plaque reduction assays have been used for the screening and evaluation of antiviral drugs [23, 24, 25]. These methods are labor intensive, time consuming, non-specific and not amendable to screen large numbers of compounds in a high-throughput manner [24, 26]. Although a new specific DENV protease activity detection system (DENPADS) was developed to monitor DENV infection and simultaneously evaluate the efficacy and cytotoxicity of potential anti-DENV candidates in cells, it is difficult to distinguish between the specific inhibitory activity of the compound on DENV protease and cytotoxicity effects on cellular functions [27]. As such, development of robust screening methods and the ability to evaluate large libraries of compounds are essential to rapidly identify new antiviral drugs against DENV. AlphaScreen^®^ is a bead-based proximity assay technology developed to measure analytes using homogenous protocol [28]. In this preliminary study, we have developed a NS2B/NS3 protease inhibition assay using AlphaScreen^®^ beads and followed by screening of the synthesized thioguanine derivatives for their possible anti-dengue activities.

## 2 Materials and methods

### 2.1 Reagents

Aprotinin, 6-thioguanine (2-amino-9H-purine-6-thiol) and other chemicals used in the chemical synthesis were purchased from Sigma-Aldrich (Milwaukee, WI, USA). AlphaScreen^®^ Detection Kit containing StrepTactin^®^ donor beads (5 mg/ml), 10x buffer and nickel chelate acceptor beads (5 mg/ml) and white 384-well Opti-plate were purchased from Perkin Elmer (Santa Clara, CA, USA). BOC-Gly-Arg-Arg-AMC was purchased from Nacalai Tesque (Kyoto, Japan). Silica gel F_254_ aluminium plate and silica gel F_254_ glass plate were purchased from Merck (Darmstadt, Germany). Peptide substrate (GGGFKEFAAGRKSLTLNLITEGGG) was synthesized and undergone quality control analysis using Mass Spectrometry and HPLC by GenScript (NJ, USA). 4-fluorobenzaldehyde, 4-hydroxy-3-methoxybenzaldehyde, 3,5-dihydroxybenzaldehyde, and deuterated dimethylsulfoxide (DMSO-D_6_) were purchased from Acros Organic (Geel, Belgium). HEPES buffer, NaOH and NaCl solutions were purchased from Fisher Scientific (Geel, Belgium). Panduratin A, a natural compound was isolated as previously described [21].

### 2.2 Expression, purification and activity of NS2B/NS3 protease

Dengue NS2B/NS3 protease enzyme was expressed and purified as previously described [22]. Briefly, NS2B/NS3 expression plasmid was propagated in *Escherichia coli* strain XL 1-Blue MRF (Agilent Technologies, Santa Clara, CA).The enzyme was isolated and purified using Ni^2+^-NTA (nickel-nitrilotriacetic acid) resin-based column. The specific proteolytic activity of NS2B/NS3 protease was determined using similar formula as published for other proteases [29, 30]. Protease assay using fluorogenic peptide susbtrate (BOC-Gly-Arg-Arg-AMC) was also used to determine the NS2B/NS3 inhibition activities of the controls and derivatives [22]. Briefly, 100 μM of fluorogenic peptide substrate and 0.57 μM protease enzyme in 200 mM Tris-HCl buffer (pH 8.5) were incubated with various concentrations of inhibitors for 1 h at 37 °C. Cleavage of the substrate by NS2B/NS3 protease and detection of the fluorescence signal were determined using Envision 2104 Multilabel Plate Reader (Perkin Elmer, Boston, MA, USA) at excitation and emission wavelength of 385 nm and 465 nm, respectively. Three independent experiments were carried out.

### 2.3 Molecular docking of model compounds as potential NS2B/NS3 protease inhibitors

Based on an initial screening of NCI diversity chemical library, a NCI compound known as diversity 0713 was found to be potentially active against DENV2 NS2B/NS3 protease. Diversity 0713 exhibits thioguanine scaffold, hence, fourteen derivatives were modeled *in silico*. These model compounds were designed by adding substituents at various positions (i.e., positions 1, 5, 7) of the thioguanine ring. Briefly, the structures of compounds were constructed using ACD/ChemSketch Freeware Version 11.01 (Advanced Chemistry Development, Inc., Canada) and optimized using PRODRG server (http://davapc1.bioch.dundee.ac.uk/prodrg/) [31]. The homology model of NS2B/NS3 protease in complex with the peptide substrate developed by Wichapong et al. was used in this *in silico* study [18]. Molecular docking simulation was carried out using the AutoDock 4.2.6 software package [32] with the docking files prepared using the AutoDockTools (ADT) 1.5.6 RC3 package. A grid map of 80 × 80 × 80 points at the position of 20, 40, -1 in the x, y, z coordinates, with a spacing of 0.375 Å between the grid points was set. The Lamarckian genetic algorithm (LGA) was used for the ligand conformational search and 100 independent docking runs were applied for diversity 0713, panduratin A and thioguanine derivatives, respectively. The docked conformations of the ligands were ranked into clusters based on the increasing order of the binding free energy. The molecular interactions of the ligands with NS2B/NS3 complex were analyzed using Accelryls^®^ Discovery Studio Visualizer 3.5 (Accelrys, Inc., San Diego, CA, USA). Parameters such as hydrogen bonding and hydrophobic interactions with NS2B/NS3 residues, free energy of binding (FEB) and estimated inhibition constant (K_*i*_) of these compounds were compared with diversity 0713 and panduratin A.

### 2.4 Synthesis of thioguanine derivatives

The synthesis of derivatives 1–11 and 12–14 was carried out as described by Salvatore and co-workers and Panneerselvam and co-workers, respectively [33, 34]. ^1^H and ^13^C-NMR spectra were determined using BrukerAvance 500 spectrometer (Bruker, USA) with TMS as an internal standard. Mass spectra were determined on XEVO-G2 QTOF#YCA153 (Waters, Milford, USA). The 3D X-ray crystal structures were resolved on Bruker SMART APEXII CCD (Bruker, USA). The melting points were obtained from an electrothermal melting point apparatus. The synthesis and characterization of thioguanine derivatives are as below:

**9-ethyl-6-(ethylthio)-9H-purin-2-amine (1).** Compound 2-amino-9H-purine-6-thiol (0.1 g, 0.6 mmol) and Cs_2_CO_3_ (0.4 g, 1.3 mmol) were dissolved in DMF (3.5 mL) and a mixture containing ethyl bromide (0.2 mL, 2.6 mmol), *tetra*-butylammonium iodide (TBAI) (0.5 g, 1.3 mmol) and DMF (3.5 mL) was added in small portions. The mixture was allowed to stand for 4 h at room temperature, then poured into water (70 mL) and extracted with ethyl acetate (2 × 50 mL). The combined organic layers were washed with water, dried over MgSO_4_ and evaporated *in vacuo*. The residual crude product was chromatographed on silica gel with *n*-hexane: ethyl acetate (1:3) to obtain **1** as a colorless amorphous (0.03 g, 19%), ^1^H-NMR (CDCl_3_): δ_H_ 1.41 (t, 3H, *J* = 7.5 Hz, 14-H_3_), 1.48 (t, 3H, *J* = 7.5 Hz, 11-H_3_), 3.31 (q, 2H, *J* = 7.5 Hz, 13-H_2_), 4.11 (q, 2H, *J* = 7.5 Hz, 10-H_2_), 4.87 (br, s, 2H, NH_2_), 7.65 (*s*, 1H, 2-H); 13C NMR (DMSO-d_6_): δ_C_ 15.5 (C-14), 15.6 (C-11), 22.3 (C-10), 38.3 (C-13), 124.6 (C-4), 140.7 (C-2), 151.1 (C-9), 159.7 (C-7), 159.96 (C-5); QTOF-MS m/z calcd for C_9_H_14_N_5_S [M+H]^+^224.3059.

**9-isopropyl-6-(isopropylthio)-9H-purin-2-amine (2).** Compound 2-amino-9H-purine-6-thiol (0.2 g, 1.2 mmol) and Cs_2_CO_3_ (0.9 g, 2.6 mmol) were dissolved in DMF (7 mL) and a mixture containing isopropyl bromide (0.5 mL, 5.2 mmol), *tetra*-butylammonium iodide (TBAI) (1.0 g, 2.6 mmol) and DMF (7 mL) was added in small portions. The mixture was allowed to stand for 4 h at room temperature, then poured into water (70 mL) and extracted with ethyl acetate (2 × 50 mL). The combined organic layers were washed with water, dried over MgSO_4_ and evaporated *in vacuo*. The residual crude product was chromatographed on silica gel with *n*-hexane: ethyl acetate (1:3) to obtain **2** as a colorless amorphous (0.05 g, 31%), ^1^H NMR (CDCl_3_): δ_H_ 1.45 (d, 6H, *J* = 6.5 Hz, 14-H_3_ and 17-H_3_), 1.55 (d, 6H, *J* = 7.0 Hz, 11-H_3_ and 16-H_3_), 4.28 (m, 1H, *J* = 7.0 Hz, 13-H), 4.67 (m, 1H, *J* = 7.0 Hz, 10-H), 4.84 (br, s, 2H, NH_2_), 7.69 (s, 1H, 2-H); 13C NMR (CDCl_3_): δ_C_ 22.5 (C-14 and C-17), 23.3 (C-11 and C-16), 30.9 (C-13), 34.2 (C-10), 46.5 (C-4), 125.9 (C-2), 137.7 (C-9), 158.3 (C-7), 167.3 (C-5); QTOF-MS m/z calcd for C_11_H_17_N_5_NaS [M+Na]^+^274.3404.

**6-(-2-ethylhexylthio)-9-(-2-ethylhexyl)-9H-purin-2-amine (3).** Compound 2-amino-9H-purine-6-thiol **(**0.2 g, 1.2 mmol) and Cs_2_CO_3_ (0.9 g, 2.6 mmol) were dissolved in DMF (7 mL) and a mixture containing 2-ethylhexyl bromide (0.9 mL, 5.3 mmol), *tetra*-butylammonium iodide (TBAI) (1.0 gram, 2.6 mmol) and DMF (7 mL) was added in small portions. The mixture was allowed to stand for 24 h at room temperature, then poured into water (70 mL) and extracted with ethyl acetate (2 × 50 mL). The combined organic layers were washed with water, dried over MgSO_4_ and evaporated *in vacuo*. The residual crude product was chromatographed on silica gel with *n*-hexane: ethyl acetate (1:3) to obtain **3** as a colorless amorphous (0.1 g, 31%), Colorless amorphous, ^1^H NMR (CDCl_3_): δ_H_ 0.86–0.95 (m, 12H, 22-H, 15-H, 27-H and 25-H), 1.26–1.33 (m, 12H, 19-H, 12-H, 20-H, 13-H, 26-H and 24-H), 1.65–1.73 (m, 1H, 11-H), 1.84–1.91 (m, 1H, 18-H), 3.37–3.49 (m, 2H, 17a-H and 10a-H), 3.88–4.00 (d, 2H, *J* = 7.0 Hz, 17b-H and 10b-H), 4.81 (br, s, 4H, NH_2_), 7.58 (s, 1H, 2-H); 13C NMR (CDCl_3_): δ_C_ 10.4 (C-27), 11.1 (C-25), 14.0 (C-22), 14.1 (C-15), 22.8 (C-21), 22.9 (C-14), 23.6 (C-24), 25.8 (C-26), 28.4 (C-20), 28.9 (C-13), 30.2 (C-12), 30.9 (C-19), 32.2 (C-11), 32.6 (C-18), 39.3 (C-17), 39.5 (C-10), 125.7 (C-4), 140.2 (C-2), 150.7 (C-9), 158.7 (C-7), 161.9 (C-5).

**2-amino-9-cyclopentyl-9H-purine-6-thiol (4).** Compound 2-amino-9H-purine-6-thiol (0.2 g, 1.2 mmol) and Cs_2_CO_3_ (0.9 g, 2.6 mmol) were dissolved in DMF (7 mL) and a mixture containing cyclopentyl bromide (0.2 mL, 2.2 mmol), *tetra*-butylammonium iodide (TBAI) (1.0 g, 2.6 mmol) and DMF (7 mL) was added in small portions. The mixture was allowed to stand for 4 h at room temperature, then poured into water (70 mL) and extracted with ethyl acetate (2 × 50 mL). The combined organic layers were washed with water, dried over MgSO_4_ and evaporated *in vacuo*. The residual crude product was chromatographed on silica gel with *n*-hexane: ethyl acetate (1:3) to obtain **4** as white powder (0.2 g, 62%), mp 160-162 °C, ^1^H NMR (CDCl_3_): δ_H_ 1.60–1.70 (m, 4H, 13-H and 14-H), 1.81 (m, 2H, 12-H), 2.02 (m, 2H, 15-H), 4.32 (m, 1H, 11-H), 4.92 (br, s, 2H, NH_2_), 7.78 (s, 1H, 2-H); 13C NMR (CDCl_3_): δ_C_ 24.9 (C-13 and C-14), 33.6 (C-12 and C-15), 42.0 (C-11), 124.5 (C-4), 137.5 (C-2), 150.8 (C-9), 158.9 (C-7), 162.9 (C-5).

**9-cyclopentyl-6-(cyclopentylthio)-9H-purin-2-amine (5).** Compound 2-amino-9H-purine-6-thiol (0.1 g, 0.6 mmol) and Cs_2_CO_3_ (0.4 g, 1.3 mmol) were dissolved in DMF (3.5 mL) and a mixture containing cyclopentyl bromide (0.3 mL, 2.6 mmol), *tetra*-butylammonium iodide (TBAI) (0.5 g, 1.3 mmol) and DMF (3.5 mL) was added in small portions. The mixture was allowed to stand for 24 h at room temperature, then poured into water (70 mL) and extracted with ethyl acetate (2 × 50 mL). The combined organic layers were washed with water, dried over MgSO_4_ and evaporated *in vacuo*. The residual crude product was chromatographed on silica gel with *n*-hexane: ethyl acetate (1:3) to obtain **5** as white powder (0.1 g, 72%), mp 129-131 °C, ^1^H NMR (CDCl_3_) δ_H_ 1.67–1.70 (m, 4H, 18-H and 19-H), 1.70–1.80 (m, 4H, 12-H and 13-H), 1.91–2.21 (m, 4H, 17-H and 20-H), 2.21–2.26 (m, 4H, 11-H and 14-H), 4.71–4.76 (m, 1H, 16-H), 4.77–4.98 (m, 1H, 10-H), 4.83 (s, 2H, NH_2_), 7.67 (s, 1H, 2-H); 13C NMR (CDCl_3_): δ_C_ 23.8 (C-12 and C-13), 24. 9 (C-18 and C-19), 32.6 (C-11 and C-14), 33.6 (C-17 and C-20), 55.3 (C-16 and C-10), 126.0 (C-4), 138.0 (C-2), 150.5 (C-9), 158.7 (C-7), 162.5 (C-5); QTOF-MS m/z calcd for C_15_H_22_N_5_S [M+H]^+^304.4336.

**Methyl** {**[2-amino-9-(2-methoxy-2-oxoethyl)-9H-purin-6-yl]sulfanyl**} **acetate (6).** Compound **(**2-amino-9H-purine-6-thiol (0.2 g, 1.2 mmol) and Cs_2_CO_3_ (0.4 g, 1.3 mmol) were dissolved in DMF (3.5 mL) and a mixture containing methyl bromoacetate (0.3 mL, 2.6 mmol), *tetra*-butylammonium iodide (TBAI) (0.5 g, 1.3 mmol) and DMF (3.5 mL) was added in small portions. The mixture was allowed to stand for 24 h at room temperature, then poured into water (70 mL) and extracted with ethyl acetate (2 × 50 mL). The combined organic layers were washed with water, dried over MgSO_4_ and evaporated *in vacuo*. The residual crude product was chromatographed on silica gel with *n*-hexane: ethyl acetate (1:3) to obtain **6** as white powder (0.2 g, 58%), mp 139-141 °C, ^1^H NMR (CDCl_3_) δ_H_ 3.75 (s, 3H, 19-H_3_), 3.79 (s, 3H, 13-H_3_), 4.09 (s, 2H, 16-H_2_), 4.82 (s, 2H, 10-H_2_), 4.89 (br, s, 2H, NH_2_), 7.69 (s, 1H, 2-H); 13C NMR (CDCl_3_): δ_C_30.7 (C-19), 43.7 (C-13), 52.7 (C-16), 52.9 (C-10), 124.9 (C-9), 140.3 (C-2), 151.0 (C-4), 158.9 (C-6), 159.5 (C-9), 167.5 (C-11), 169.7 (C-17); QTOF-MS m/z calcd for C_17_H_14_N_5_O_4_S [M+H]^+^312.3249.

**6-{[(pyridin-2-yl)methyl]sulfanyl}oolpl.-9H-purin-2-amine (7).** Compound 2-amino-9H-purine-6-thiol (0.1 g, 0.6 mmol) and Cs_2_CO_3_ (0.4 g, 1.3 mmol) were dissolved in DMF (3.5 mL) and a mixture containing methyl pyridinium bromide hydrobromide (0.3 g, 1.3 mmol), *tetra*-butylammonium iodide (TBAI) (0.5 g, 1.3 mmol) and DMF (3.5 mL) was added in small portions. The mixture was allowed to stand for 24 h at room temperature, then poured into water (70 mL) and extracted with ethyl acetate (2 × 50 mL). The combined organic layers were washed with water, dried over MgSO_4_ and evaporated *in vacuo*. The residual crude product was chromatographed on silica gel with *n*-hexane: ethyl acetate (1:3) to obtain **7** as white powder (0.08 g, 53%), mp 260-263 °C, ^1^H NMR (DMSO-d_6_) δ_H_ 4.65 (s, 2H, 11-H_2_), 6.46 (s, 2H, NH_2_), 7.26–7.91 (m, 4H, 17-H, 16-H, 15-H and 14-H), 8.51 (s, 1H, 2-H),12.57 (s, 1H, 1-H); 13C NMR (DMSO-d_6_): δ_C_ 33.6 (C-11), 122.7 (C-15), 123.9 (C-17), 137.2 (C-4), 137.6 (C-16), 139.3 (C-2), 141.9 (C-14), 149.6 (C-9), 152.3 (C-12), 158.3 (C-7), 160.0 (C-5).

**6-(benzylsulfanyl)-9H-purin-2-amine (8).** Compound 2-amino-9H-purine-6-thiol (0.1 g, 0.6 mmol) and Cs_2_CO_3_ (0.4 g, 1.3 mmol) were dissolved in DMF (3.5 mL) and a mixture containing benzyl bromide (0.2 mL, 1.3 mmol), *tetra*-butylammonium iodide (TBAI) (0.5 g, 1.3 mmol) and DMF (3.5 mL) was added in small portions. The mixture was allowed to stand for 12 h at room temperature, then poured into water (70 mL) and extracted with ethyl acetate (2 × 50 mL). The combined organic layers were washed with water, dried over MgSO_4_ and evaporated *in vacuo*. The residual crude product was chromatographed on silica gel with *n*-hexane: ethyl acetate (1:3) to obtain **8** as white powder (0.1 g, 74%), m.p. 193-195 °C [195 °C [1]], ^1^H NMR (DMSO-d_6_) δ_H_ 5.44 (s, 2H, 11-H_2_), 5.67 (s, 2H, NH_2_), 7.33 (m, 6H, 13-H, 17-H, 15-H, 14-H, 16-H and 2-H), 9.65 (s, 1H, 1-H); 13C NMR (DMSO-d_6_): δ_C_ 32.8 (C-11), 114.0 (C-13 and C-17), 134.2 (C-15), 134.6 (C-14 and C-16), 137.5 (C-4), 139.8 (C-12), 141.3 (C-2), 149.9 (C-9), 157.6 (C-7), 159.8 (C-5).

**9-benzyl-6-(benzylsulfanyl)-9H-purin-2-amine (9).** Compound **(**2-amino-9H-purine-6-thiol (1.0 g, 5.9 mmol) and Cs_2_CO_3_ (4.3 g, 13 mmol) were dissolved in DMF (25 mL) and a mixture containing benzyl bromide (3.1 mL, 26.3 mmol), *tetra*-butylammonium iodide (TBAI) (5.0 g, 1.3 mmol) and DMF (25 mL) was added in small portions. The mixture was allowed to stand for 24 h at room temperature, then poured into water (70 mL) and extracted with ethyl acetate (2 × 50 mL). The combined organic layers were washed with water, dried over MgSO_4_ and evaporated *in vacuo*. The residual crude product was chromatographed on silica gel with *n*-hexane: ethyl acetate (1:3) to obtain **9** as blocked crystal from methanol (1.4 g, 68%), mp 155-157 °C [157 °C [2]], ^1^H NMR (CDCl_3_): δ_H_ 4.50 (s, 2H, NH_2_), 4.85 (s, 2H, 18-H_2_), 5.16 (s, 2H, 10-H_10_), 7.41 (m, 10H, 20-H, 24-H, 12-H, 16-H, 21-H, 23-H, 13-H, 15-H, 22-H, 14-H), 7.52 (s, 1H, 2-H); 13C NMR (CDCl_3_): δ_C_ 32.6 (C-18), 46.6 (C-10), 127.2 (C-14), 127.6 (C-20 and C-24), 128.3 (C-13 and C-15), 128.5 (C-23 and C-21), 129.0 (C-12 and C-16), 129.1 (C-4), 135.6 (C-11), 137.8 (C-19), 139.9 (C-2), 150.8 (C-9), 158.9 (C-7), 161.7 (C-5).

***N*, 9-dibenzyl-6-(benzylsulfanyl)-9H-purin-2-amine (10).** Compound 2-amino-9H-purine-6-thiol (0.1 g, 0.6 mmol) and Cs_2_CO_3_ (1.4 g, 3.9 mmol) were dissolved in DMF (12.5 mL) and a mixture containing benzyl bromide (0.5 mL, 3.9 mmol), *tetra*-butylammonium iodide (TBAI) (1.5 g, 3.9 mmol) and DMF (12.5 mL) was added in small portions. The mixture was allowed to stand for 48 h at room temperature, then poured into water (70 mL) and extracted with ethyl acetate (3 × 70 mL). The combined organic layers were washed with water, dried over MgSO_4_ and evaporated *in vacuo*. The residual crude product was chromatographed on silica gel with *n*-hexane: ethyl acetate (1:3) to obtain **10** as white powder (0.07 g, 25%), mp 132-134 °C, ^1^H NMR (CDCl_3_): δ_H_ 4.45 (s, 2H, 18-H_12_), 4.63 (s, 2H, 26-H_2_), 5.14 (s, 2H, 10H_2_), 5.34 (s, 1H, NH), 7.14–7.50 (m, 15H, 20-H, 24-H, 28-H, 32-H, 12-H, 16-H, 22-H, 30-H, 14-H, 21-H, 23-H, 29-H, 31-H, 13-H and 15-H), 7.51 (s, 1H, 2-H); 13C NMR (CDCl_3_): δ_C_ 32.5 (C-18), 46.0 (C-26), 46.7 (C-10), 127.4 (C-22), 127.8 (C-30), 128.2 (C-14), 128.4 (C-20), 128.5 (C-24), 128.6 (C-28 and C-32), 128.7 (C-12 and C-16), 128.8 (C-21 and C-23), 128.9 (C-29 and C-31), 129. 0 (C-13 and C-15), 135.9 (C-11), 137.9 (C-19), 139.5 (C-27), 150.6 (C-2), 158.8 (C-9), 160.7 (C-7), 165.0 (C-5); QTOF-MS m/z calcd for C_26_H_23_N_5_S [M ]^+^437.5593, found 437.2747.

Continued elution with *n*-hexane: ethyl acetate (1:3) resulted in **11** (***N*, *N*, 9-tribenzyl-6-(benzylsulfanyl)-9H-purin-2-amine)** as a brown liquid (0.2 g, 70%), ^1^H NMR (CDCl_3_): δ_H_ 4.45 (s, 8H, 18-H, 26-H, 33-H and 10-H), 7.21–7.38 (m, 20H, 20-H – 24-H, 28-H – 32-H, 35-H – 39-H and 12-H – 16-H); 13C NMR (CDCl_3_): δ_C_ (CDCl_3_) 33.6 (C-18), 65.4 (C-26 and C-33), 72.1 (C-10), 127.6 (C-30, C-27 and C-22), 127.8 (C-24 and C-20), 127.9 (C-28, C-32, C-35 and C-39), 128. 4 (C-29, C-31, C-36 and C-38), 128.7 (C-21 and C-23), 128.8 (C-13 and C-15), 129.7 (C-12 and C-16), 129.9 (C-4), 131.6 (C-11, C-34 and C-27), 133.2 (C-19), 134.5 (C-2), 137.8 (C-9), 138.3 (C-7), 139.3(C-5); QTOF-MS m/z calcd for C_33_H_29_N_5_S [M ]^+^527.6818, found 527.3339.

**2-(4-fluorobenzylideneamino)-9H-purine-6-thiol (12).** Compound 2-amino-9H-purine-6-thiol (0.1 g, 0.6 mmol) was mixed with 4-fluorobenzaldehyde (0.06 mL, 0.6 mmol), stirred for 15 min and the liquid mixture of ethanol and 10% (v/v) NaOH [3.0 mL (1:1)] was added in small portions. The mixture was allowed to stand for 2 h at 80 °C, then neutralized with HCl (6 M) until precipitated. The solid product was filtered, washed with water followed by ethyl acetate. The residual crude product was chromatographed on silica gel with CHCl_3_: methanol (2:2) to obtain **12** as yellow crystal from methanol (0.1 g, 75%), mp 149-151 °C, ^1^H NMR (DMSO-D_6_): δ_H_ 7.32 (dd, 2H, *J* = 8.5 Hz, *J* = 4.0 Hz, 15-H and 17-H), 7.34 (s, 1H, 2-H), 7.78 (s, 1H, 12-H), 7.87 (m, 2H, 14-H and 18-H); 13C NMR (CDCl_3_): δ_C_ 116.1 (C-15 and C-17), 116.3 (C-13), 125.8 (C-14 and C-18), 130.3 (C-4), 130.4 (C-2), 131.0 (C-7), 142.1 (C-9), 163.1 (C-12), 165.1 (C-16), 188.5 (C-5); QTOF-MS m/z calcd for C_12_H_8_FN_5_S [M+H]^+^274.2976.

**2-methoxy-4-**{**[(6-sulfanyl-9H-purin-2-yl)imino]methyl**}**phenol (13).** Compound 2-amino-9H-purine-6-thiol (0.1 g, 0.6 mmol) was mixed with 4-hydroxy-3-methoxybenzaldehyde (0.09 g, 0.6 mmol), stirred up for 15 minutes and the liquid mixture of ethanol and NaOH 10% [3.0 mL (1:1)] was added in small portions. The mixture was allowed to stand for 2 h at room temperature, then neutralized with HCl (6 M) until precipitated. The solid product was filtered, washed with water followed by ethyl acetate. The residual crude product was chromatographed on silica gel with methanol (100%) to obtain **13** as white powder (0.08 g, 45%), decomps. 300 °C, ^1^H-NMR (DMSO-d_6_): δ_H_ 3.85 (s, 3H, OMe), 7.126 (br, s, 3H, 17-H, 14-H and 18-H), 8.16 (s, 1H, 2-H), 8.28 (s, 1H, 12-H), 12.59 (br, s, 1H, 1-H); 13C NMR (DMSO-d_6_): δ_C_ 84.1 (C-20), 127.5 (C-17), 133.1 (C-14), 140.3 (C-18), 155.0 (C-4), 155.1 (C-2), 155.2 (C-16), 161.1 (C-15), 161.4 (C-7), 163.2 (C-9), 168.5 (C-12), 172.4 (C-5).

**5-**{**[(6-sulfanyl-9H-purin-2-yl)imino]methyl**}**benzene-1,3-diol (14).** Compound 2-amino-9H-purine-6-thiol (0.1 g, 0.6 mmol) was mixed with 3,5-dihydroxybenzaldehyde (0.08 g, 0.6 mmol), stirred up for 15 minutes and the liquid mixture of ethanol and NaOH 10% [3.0 mL (1:1)] was added in small portions. The mixture was allowed to stand for 2 h at room temperature, then neutralized with HCl (6 M) until precipitated. The solid product was filtered, washed with water followed by ethyl acetate. The residual crude product was chromatographed on silica gel with CH_2_Cl_2_: methanol (2:2) to obtain **14** as brown powder (0.05 g, 29%), decomps. 300 °C, ^1^H NMR (DMSO-d_6_): δ_H_ 7.02 (br, s, 3H, 16-H, 14-H and 18-H), 8.14 (s, 1H, 12-H), 8.74 (s, 1H, 2-H), 12.51 (br, s, 1H, 1-H); 13C NMR (DMSO-d_6_): δ_C_ 95.6 (C-16), 97.4 (C-14 and C-18), 121.0 (C-4), 141.0 (C-13), 147.6 (C-2), 148.9 (C-7), 154.6 (C-9), 160.2 (C-15 and C-20), 170.0 (C-12), 172.0 (C-5); QTOF-MS m/z calcd for C_12_H_13_N_6_O_2_S [M+NH_4_]^+^305.3356.

### 2.5 AlphaScreen^®^ assay optimization and screening of derivatives

**Fig. 1:**
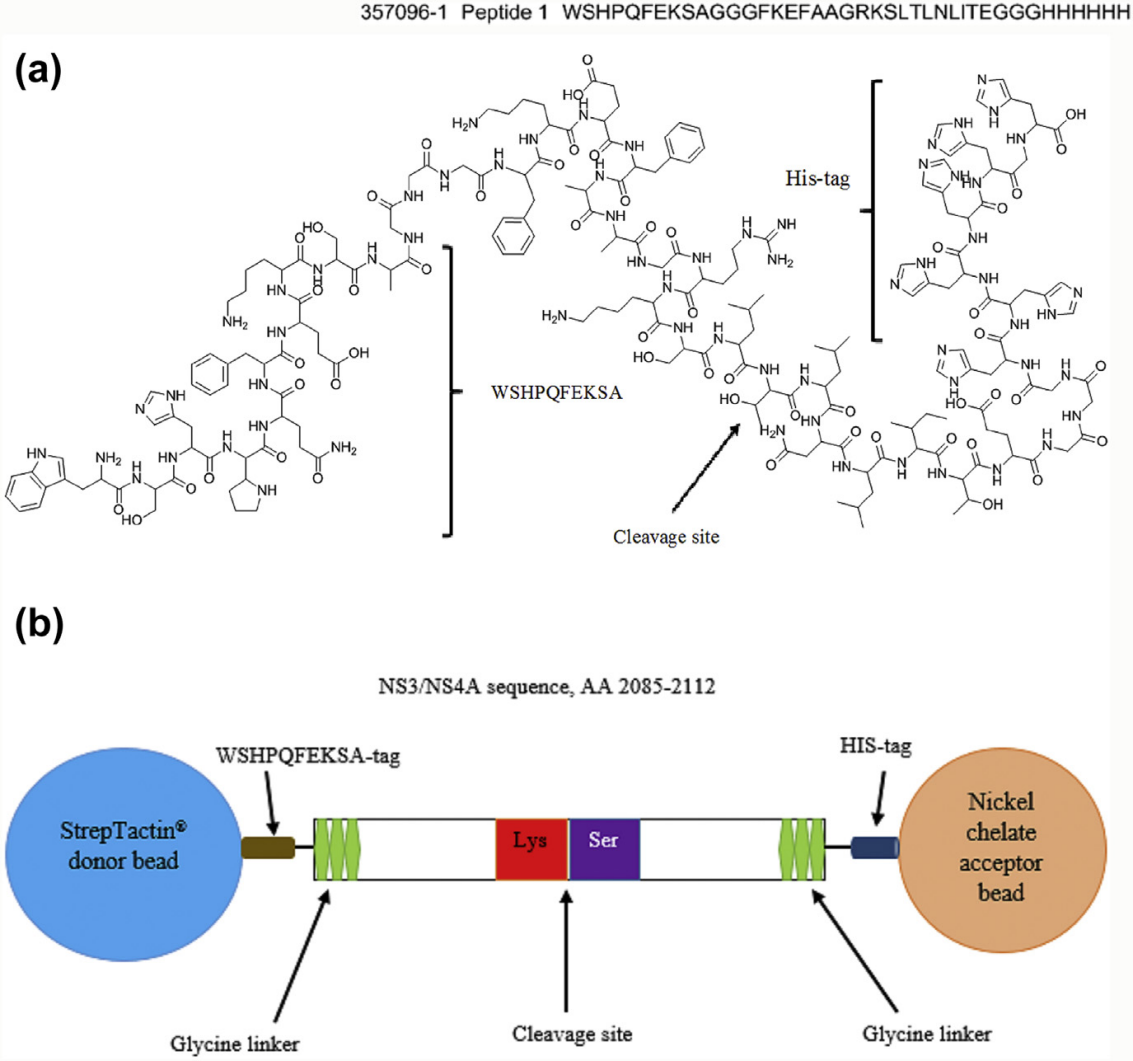
(a) The peptide substrate used in this assay is tagged with a specific sequence at N-terminal and His-tag at C-terminal (b) An illustration showing the interaction between the peptide substrate and the AlphaScreen^®^ beads.

The peptide substrate used in this assay has a known NS2B/NS3 cleavage site between lysine (K), and serine (S) (GRK **↓** SLT), and is a part of a sequence of dengue polyprotein precursor (NS3/NS4A) as reported by Khumtong and co-workers [35, 36]. The peptide substrate was tagged with a specific sequence (WSHPQFEKSA) at N-terminal and polyhistidine at C-terminal. This specific sequence has a binding affinity towards StrepTactin^®^, an engineered streptavidin [37]. Glycine linkers were added on both sides of untagged peptide substrate (Fig. 1a and b). To determine the extent of N-terminal labelling on the peptide substrate, competitive displacement assay using AlphaScreen^®^ TruHits Detection Kit (Perkin Elmer, USA) consisting of StrepTactin^®^ donor beads and biotinylated acceptor beads was carried out. On the other hand, to ensure that the peptide substrate is adequately tagged with polyhistidine on the C-terminal, competitive binding assay using Histidine (Nickel Chelate) Detection Kit (Perkin Elmer, USA) was carried out. This kit comprises of both StrepTactin^®^ donor beads and nickel chelate acceptor beads.

In general, AlphaScreen^®^ experiments were carried out in white 384-well plates (Perkin Elmer, Boston, MA, USA) in a total reaction volume of 25 μL under subdued light. All dilutions were initially performed in starting assay solution containing 25 mM HEPES buffer (pH 7.4), 100 mM NaCl and 0.01 % (w/v) BSA. StrepTactin^®^ donor beads and nickel chelate acceptor beads were used at final concentration of 20 μg/mL. The 384-well plate was read using Envision 2104 Multilabel Plate Reader (Perkin Elmer, Boston, MA, USA) according to AlphaScreen^®^ settings (excitation and emission wavelength at 680nm and 520-620nm, respectively). For optimization, peptide substrate (0.3 nM–3 μM) and NS2B/NS3 protease were cross-titrated to determine the concentrations that generate optimal AlphaScreen^®^ signals. Briefly, in each well, 5 μL of peptide substrate and NS2B/NS3 protease enzyme were mixed with 5 μL assay buffer and incubated at 37 °C. After incubating for 1 h, 5 μL of donor beads and acceptor beads were added, followed by 1 h incubation at room temperature prior to reading of signal. To develop an assay that delivers high signal and low background, different buffer system, various concentrations of HEPES, NaCl and BSA, pH and incubation time were optimized. The optimum conditions were determined based on the maximum signal obtained from well containing peptide alone and minimum signal produced by well containing peptide substrate, enzyme and beads, indicating maximum cleavage of the NS2B/NS3 enzyme on the substrate and lowest background signal. Once the assay was optimized, it was used to screen the compounds for their potential NS2B/NS3 protease inhibition activity. Aprotinin and panduratin A were used as positive controls. Since the amount obtained for diversity 0713 was in minute quantity and was insufficient for subsequent testing, diversity 0713 was not used as control in this study. All compounds were dissolved in DMSO and the assay was performed in either 1% (v/v) or 10% (v/v) DMSO in final concentrations, depending on the solubility of the thioguanine derivatives.

### 2.6 Data analysis

IC_50_ values were derived based on non-linear regression analysis computed using GraphPad Prism 5.0 (GraphPad Software, La Jolla, CA, USA). All data were presented as mean ± SD. The zʹ factor was calculated as described by Zhang and co-workers [38]. zʹ factor = 1–3×(σH+σL)/|μH−μL|, where σH and σL represent the standard deviations of the AlphaScreen^®^ signal of the substrate in the absence (high signal, H) and presence (low signal, L) of the protease, and μH and μL represent the mean values of high signal and low signal, respectively. To evaluate the robustness of the assay, interday variations were evaluated in assay plate replicates and coefficient variation (CV) was calculated using the formula; % CV = (SD/√n)/average x 100.

## 3 Results

### 3.1 Molecular docking

**Fig. 2:**
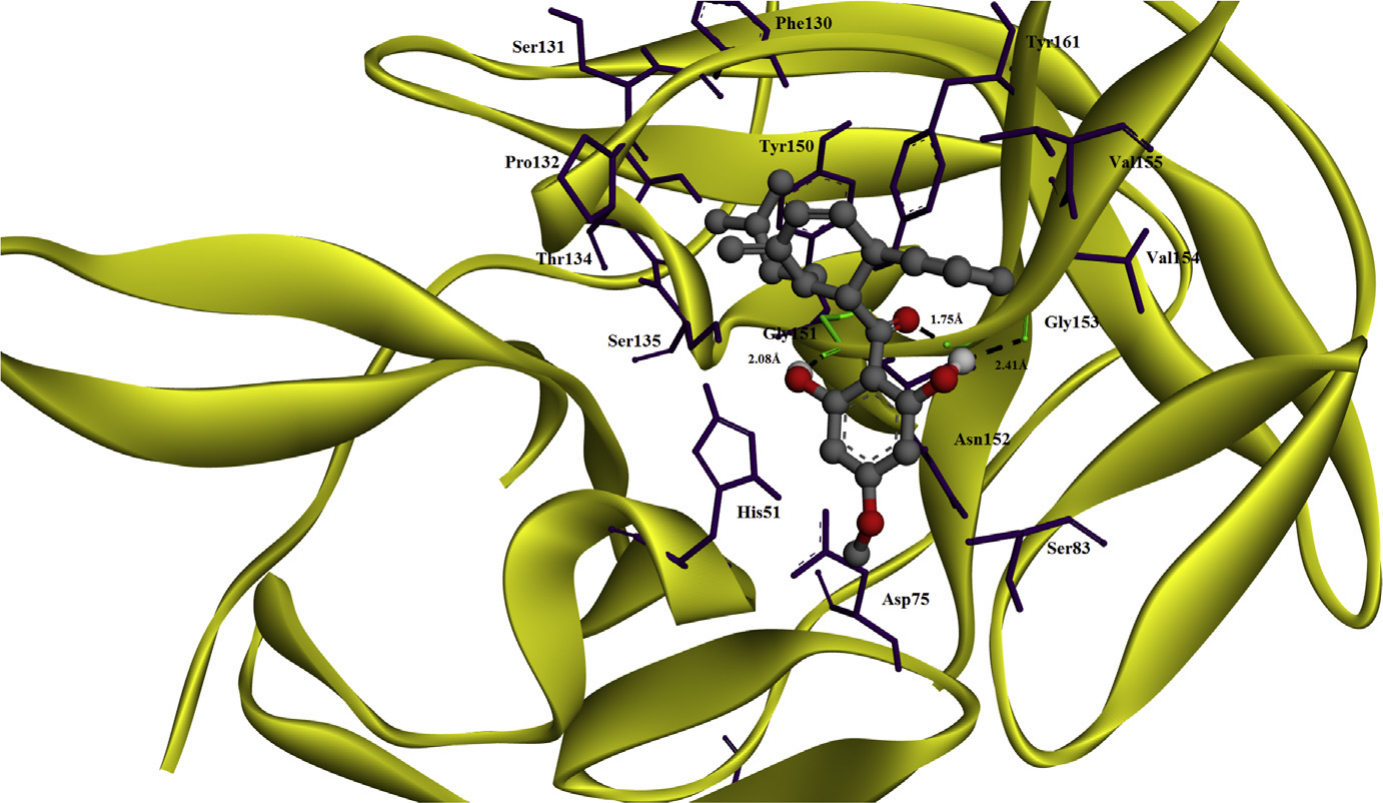
Molecular docking of panduratin A in the active site of the serine protease domain of the NS2B/NS3 protease. The conformation of compounds is shown in ball and stick representation. Atoms are colored grey for carbon, blue for nitrogen, brown for sulfur, red for oxygen and white for hydrogen. Hydrogen bonds are depicted as a green line. NS2B/NS3 protease is represented as yellow ribbon. Residues labeled in purple are those that involved in hydrophobic interactions, while those in green are involved in hydrogen bonding and hydrophobic interactions.

Docking simulation of diversity 0713 compound within the active site of DENV2 NS2B/NS3 protease showed an interaction with N152, a sub-pocket 2 residue. This compound was also observed to interact with G151 and G153 by forming hydrogen bonds and Y150 and Y161 via hydrophobic bonds. The calculated FEB and the estimated inhibition constant (K_i_) for diversity 0713 was −6.64 kcal/mol and 13.49 μM, respectively (Table 1). On the other hand, docking of panduratin A within the active site of dengue NS2B/NS3 protease revealed that it interacts with G151 and G153 of the NS2B/NS3 protease, similar to the previous work carried out by Lee and co-workers (Fig. 2) [39]. Panduratin A also has similar interactions as diversity 0713, such as hydrogen bonds formation with G151 and G153 and hydrophobic bonds with Y150 and Y161 residues, respectively. However, the calculated FEB and K_i_ for panduratin A were slightly lower than diversity 0713 compound at -6.93 kcal/mol and 8.36 μM, respectively, indicating that panduratin A may probably have similar inhibitory activity as diversity 0713 compound.

**Table 1:** Summary of molecular interactions of panduratin A and 14 derivatives (100 docking runs).

Compound	NS2B/NS3 protease residue										FEB (kcal/mol)	Estimated Inhibition Constant, K_*i*_ (μM)
	Hydrogen bonding					Hydrophobic interactions						
	S1	S2	S3	S4	Others	S1	S2	S3	S4	Others		
Panduratin A					G151, G153, D81, G82	S135(2), Y150, Y161(5)	D75, N152(7)		V154(2)	H51(2), D81, G82(2), S83, M84, F130, G151(3), G153(8), V155(2)	-6.93	8.36
Diversity 0713		N152			G151, G153	Y150, Y161					-6.64	13.49
1	S135				F130, S131	D129(3), S135(2), Y150(4), Y161(7)				F130(4), S131(2), P132(3), T134(3), G151(2)	-4.80	303.19
2		N152			G151, G153	S135(3), Y150, Y161(9)	N152(6)		V154(2)	H51, P132, G151(3), G153(4), V155(2)	-5.51	91.38
3		N152			G151, G153	135(3), Y150(1), Y161(8)	N152(6)		V154	H51, M84, I86, F130(3), S131(2), P132(3), G151(6), G153(7), 155(4)	-6.37	21.49
4		N152			G151, G153	S135(2), Y150, Y161(8)	N152(5)			H51, F130(2), S131, G151(7), G153(3)	-6.03	37.89
5		N152			G151, G153	S135(2), Y150(2), Y161(8)	N152(6)		V154(2)	H51, M84, F130(3), S131, T134, G151, G153(5), V155(2)	-7.52	3.08
6	S135	N152			G151, G153, V155	S135, Y150, V154(3), Y161(8)	N152(6)			M84, S131, P132, G151(4), G153(5), V155(2)	-5.06	195.64
7		N152			G151, G153, P132	S135(2), Y150(2), Y161(8)	N152(4)		V154(2)	F130(2), S131(2), P132(2), T134(2), G151(2), G153(7), V155(2)	-6.42	19.72
8		N152			G151, G153	S135(2), Y150(2), Y161(9)	N152(5)		V154(2)	P130(3), S131(3), P132(2), T134(2), G151(2), G153(7), V155(2)	-6.79	10.54
9		N152			G151, G153	S135, Y150(3), Y161(7)	N152 (5)		V154	S83(2), M84(2), P130(3), S131(3), P132(2), T134(2), G151, G153(7), V155	-7.10	6.29
10	S135					S135 (5), Y150, Y161(7)	N152			I36(2), V52, F130, S131, P132(4), G151(5), G153(4), V155(2)	-7.53	3.02
11	Y161				G151	S135(5), Y150(2), Y161(6)	D75(3), N152			I36(2), W50(2), H51(16), V52, V72(2), D81(2), G82(2), F130(2), S131(2), P132(4), T134, G151(2), G153, V155	-8.88	0.311
12	Y161				G151, G153, W50, G82, M84	Y161	D75(2), N152(7)			W50(3), H51, V72(4), D81(7), G82, S83, M84, G151(3), G153(3)	-6.90	8.70
13	Y161				G151, G153, W50, G82, M84	Y161	D75(2), N152(6)			W50 (5), V72 (4), D81 (8), G82, S83 (2), M84, G151 (3), G153	-6.51	16.86
14	S135, D129				G153, F130, T134	S135(3), Y150(4), Y161(7)	N152(6)			S83(2), M84, F130(3), S131(2), T134(2), G151(4), G153(5)	-6.94	8.16

**Sub-pocket residues**: **S1** (D129, S135, Y150, Y161), **S2** (D75, D82, G83, N84, N152), **S3** (F85, Q86, L87), **S4** (V154), **Catalytic triad** (H51, D75, S135). *Number in bracket ( ) represent the number of hydrophobic interactions formed.

On the other hand, thioguanine derivatives exhibited binding interactions with important residues such as sub-pockets (1 and 2) of dengue NS2B/NS3 protease. Nine out of fourteen compounds (Compounds 1, 2, 3, 4, 6, 7, 8, 12, and 13) have FEB and estimated K_i_ values higher than panduratin A. Compounds 5, 9, 10, 11 and 14 have lower FEB and K_i_ values as compared with panduratin A, suggesting greater inhibitory activity against the dengue protease (Table 1). Generally, the molecular interactions of these derivatives with the protease have some similarity with panduratin A, for example most compounds form hydrogen bonding and/or hydrophobic interactions with G151 and G153 residues and have hydrophobic interactions with at least one of the catalytic triad residues (H51, D75, and S135). Brinkworth and co-workers have stressed the importance of G153 residues in overall substrate binding [40]. Only compounds 1, 6, 10 and 14 forms hydrogen bonds with at least one of the catalytic triad residues (H51, D75, and S135). These residues are important because they are involved in the cleavage of the viral polyprotein. In addition, most of the model compounds were demonstrated to interact with N152, one of the residues found in the sub-pocket 2. None of the compounds were observed to interact with residues in sub-pocket 3 (F85, Q86 and L87) or sub-pocket 4 (V154). Compound 11 scored the lowest FEB and K_*i*_ value but its binding orientation is slightly different from panduratin A as it interacts with both G151 and Y161 (sub-pocket 1). However, both compounds have hydrophobic interactions with all three catalytic triad residues (Table 1). Based on the molecular docking simulation, all fourteen model compounds were found to be potentially active against NS2B/NS3 protease; hence, these compounds were synthesized for further evaluation.

### 3.2 Synthesis and characterization of thioguanine derivatives

**Fig. 3:**
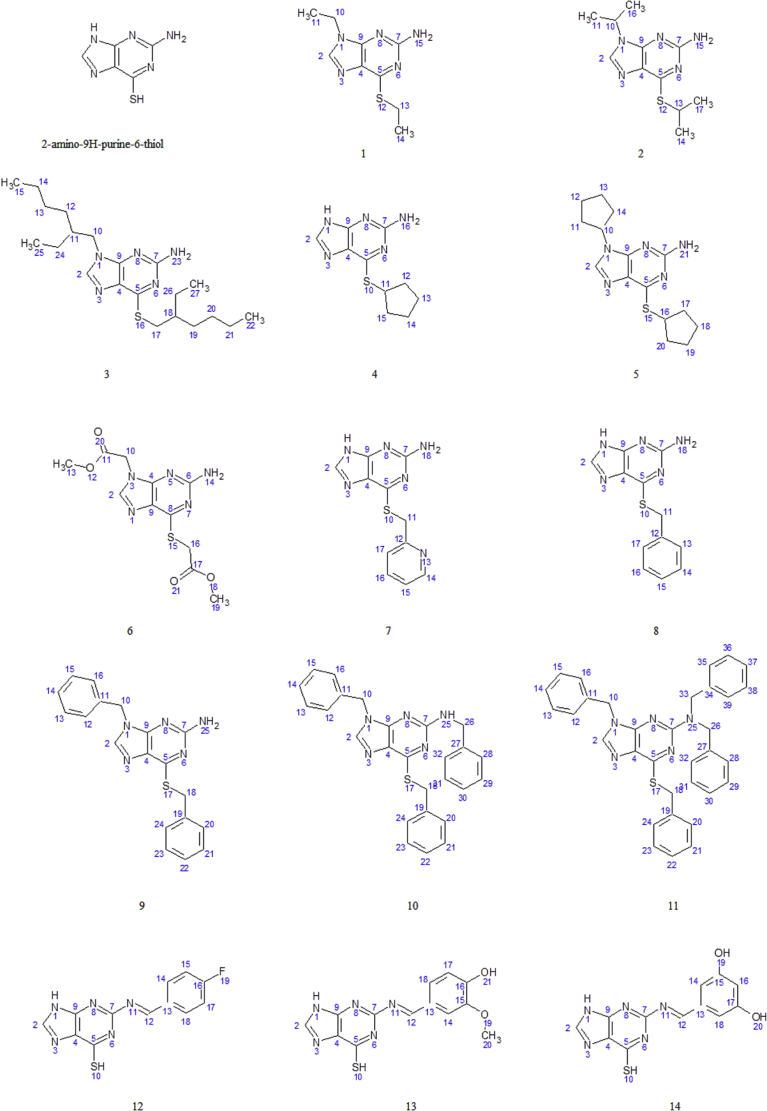
The structures of the synthesized thioguanine derivatives.

The synthesis of thioguanine derivatives was divided into 2 types of reaction, alkylation (*N*-alkylation and *S*-alkylation) and Schiff base formation. The alkylation was carried out by reacting thioguanine and alkyl bromide. In this reaction, the chemo selective *S*-alkylation (4, 7 and 8) was applied by using tetrabutyl ammonium iodide (TBAI) as phase transfer catalyst (PTC) while cesium carbonate (Cs_2_CO_3_) was used as the base catalyst. A polar aprotic solvent such as dimethylformamide (DMF) was employed as the solvent in the *N*-alkylation. Further alkylation at NH (1-3, 5, 6 and 9) as well as NH_2_ (10 and 11) required at least 2-fold of alkyl bromide mole number. The second series of the thioguanine derivatives (12-14) were synthesized based on the reaction between primary amine of thioguanine and a diverse aromatic aldehyde in a mixture of NaOH-ethanol solution. The structures of the synthesized compounds are shown in Fig. 3.

### 3.3 Optimization of the assay

**Fig. 4:**
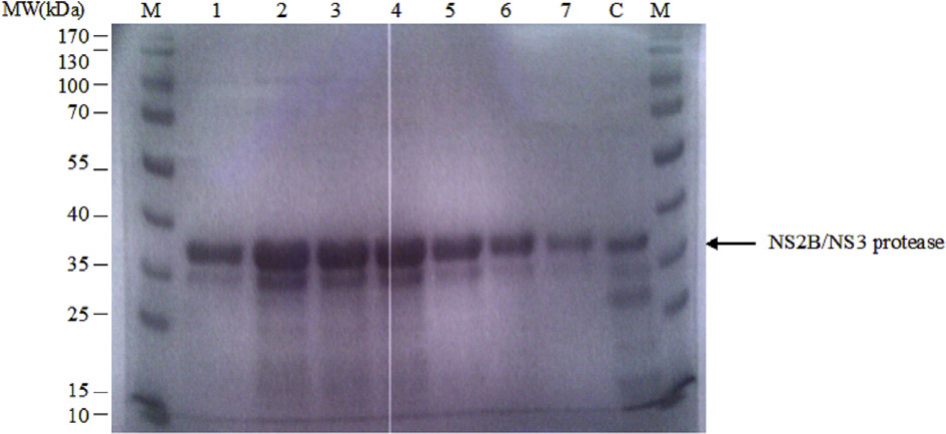
SDS-PAGE of fractions obtained from purification of DENV2 protein precursor on Ni2+-NTA column; Lane M: protein molecular weight marker; Lane 1, 2, 3, 4, 5, 6 and 7 are the fractions eluted from Ni2+-NTA column; Lane C is control.

**Fig. 5:**
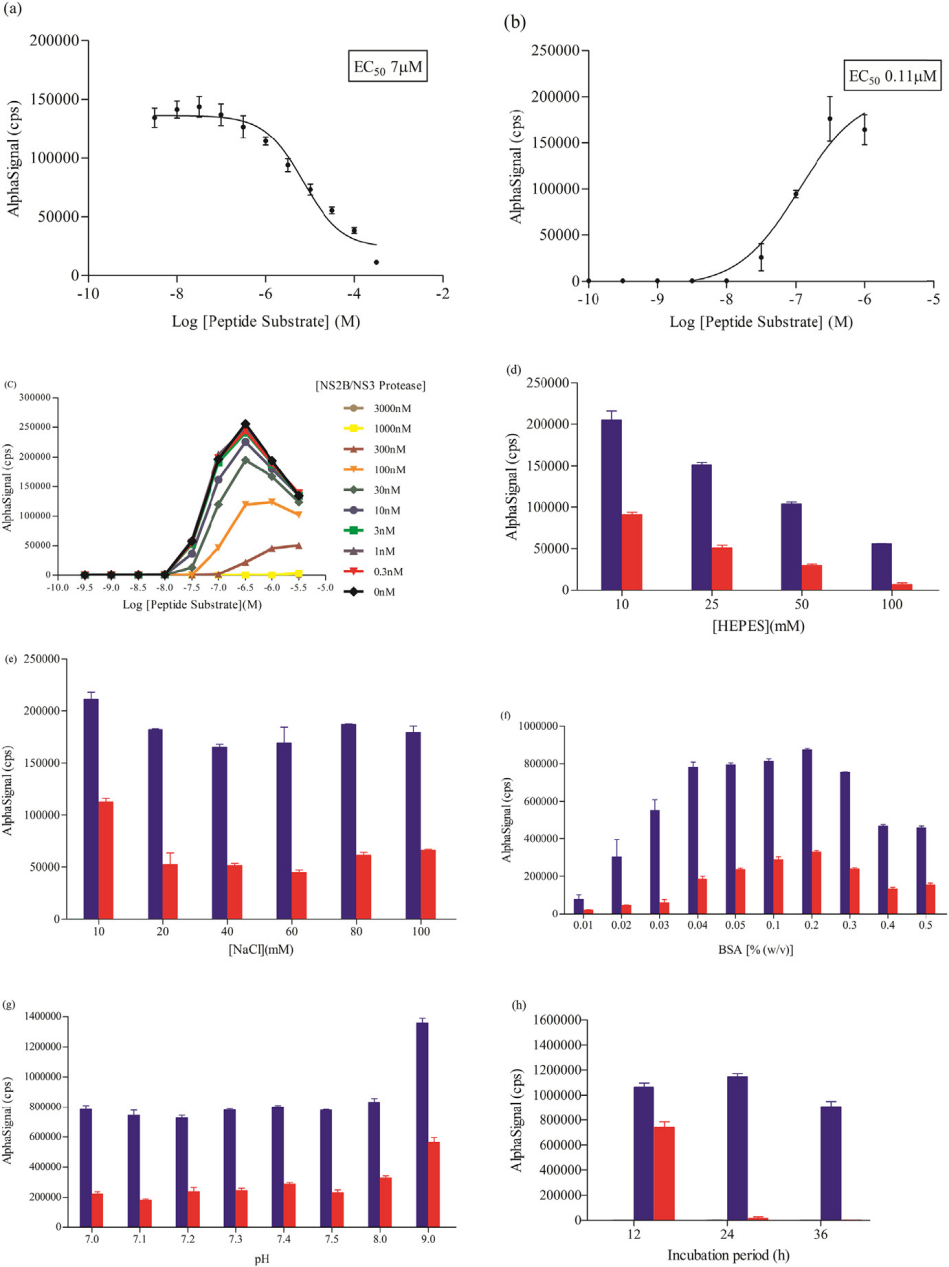
Titration curve of the competitive displacement assay to determine the extent of labelling on the peptide substrate. (a) Displacement of biotinylated acceptor beads from the StrepTactin^®^ donor beads by the peptide substrate (b) Bridging of the the StrepTactin^®^ donor beads and the nickel-chelated donor beads by the peptide substrate, increasing the concentration of substrate results in an increase of signal (c) Cross-titration curve of the peptide substrate and NS2B/NS3 protease which a hook point was reached at 300 nM of peptide substrate. (d) Optimization of HEPES concentration (e) Optimization of NaCl concentration (f) Optimization of BSA concentration (g) Optimization of pH of assay solutions (h) Optimization of incubation time. All data are presented as mean alphasignal ± SEM of triplicates of a single independent experiment. Buffer-Yellow; Peptide-Purple; Peptide and enzyme-Red.

The NS2B/NS3 protease enzyme was expressed and purified as described previously (Fig. 4). The specific activity of the expressed NS2B/NS3 protease enzyme was determined at 12 mU/mg and the concentration of the protease was 0.6 mg/ml. A typical sigmoidal curve was observed in the TruHits competitive assay indicating displacement of biotinylated acceptor beads from the donor beads by the peptide substrate (Fig. 5a). Displacement of the biotinylated acceptor beads at low substrate concentrations indicated that the substrate was adequately tagged with WSHPQFEKSA sequence (EC_50_ value at 7 μM). Similarly, the extent of polyhistidine tagging on the substrate was determined and based on the titration curve, signal increased proportionally to the amount of peptide substrate present, as shown in Fig. 5b, indicating that the substrate was adequately bound onto nickel chelate acceptor beads at low concentrations (EC_50_ value at 0.11 μM).

Cross titration of peptide substrate and NS2B/NS3 protease revealed a typical bell-shaped curve illustrating the hook effect when the concentration of peptide substrate was greater than 300 nM. Beyond this point, saturation of the peptide substrate with the beads caused a decrease in signal intensity. In addition, increasing concentration of enzyme produced a reduction in signal, indicating proteolytic activity of the NS2B/NS3 enzyme. A significant decrease in signal was observed from 100 nM onwards. The peptide substrate was almost completely cleaved in the presence of 3 μM enzyme (Fig. 5c). Based on optimal conditions, 100 nM of NS2B/NS3 enzyme and 300 nM of substrate were used in subsequent assays.

Initially, a starting buffer consisting of 25 mM HEPES, 100 mM NaCl, 0.01% (w/v) BSA, pH 7.4) was used. However, the assay buffer was re-optimized to increase signal/background (S/B) ratios. Eventually, 10 mM Hepes (pH 9.0), 20 mM NaCl and 0.2% BSA provided the most robust assay conditions. The addition of BSA improved the signal by a few hundred thousand counts per second (Fig. 5d–g). To evaluate the protein digestion duration, a time course experiment was carried out and based on the results, a 24 h incubation time was found to be optimal. At this point, the proteolytic activity was the highest (lowest signal in the presence of enzyme) and background signal generated was also the lowest (Fig. 5h).

### 3.4 Screening of thioguanine derivatives using AlphaScreen^®^ assay

**Fig. 6:**
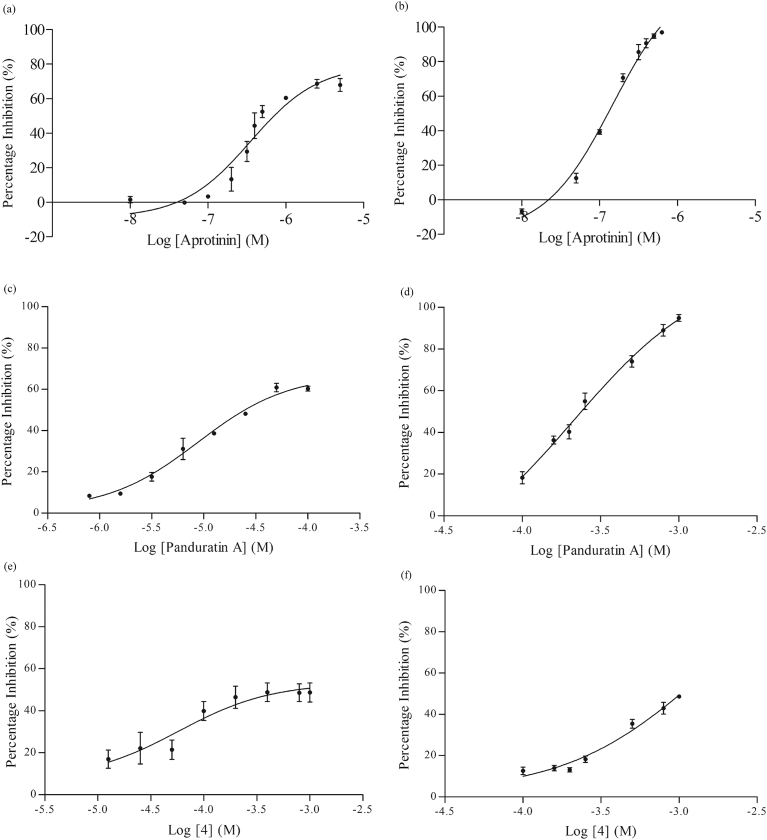
(a) The effect of aprotinin on the NS2B/NS3 protease activity using AlphaScreen^®^ assay (IC_50_ value at 0.35 μM) (b) The effects of aprotinin on the NS2B/NS3 protease activity using protease assay (IC_50_ value at 0.15 μM) (c) The effect of panduratin A on the NS2B/NS3 protease activity using AlphaScreen^®^ assay (IC_50_ undetermined) (d) The effect of panduratin A on the NS2B/NS3 protease activity using protease assay (IC_50_ value at 211.5 μM) (e) The effect of compound 4 on the NS2B/NS3 protease activity using AlphaScreen^®^ assay (IC_50_ undetermined) (f) The effect of compound 4 on NS2B/NS3 protease activity using protease assay (IC_50_ undetermined). Data are presented as mean alphasignal ± SEM of three independent experiments.

**Fig. 7:**
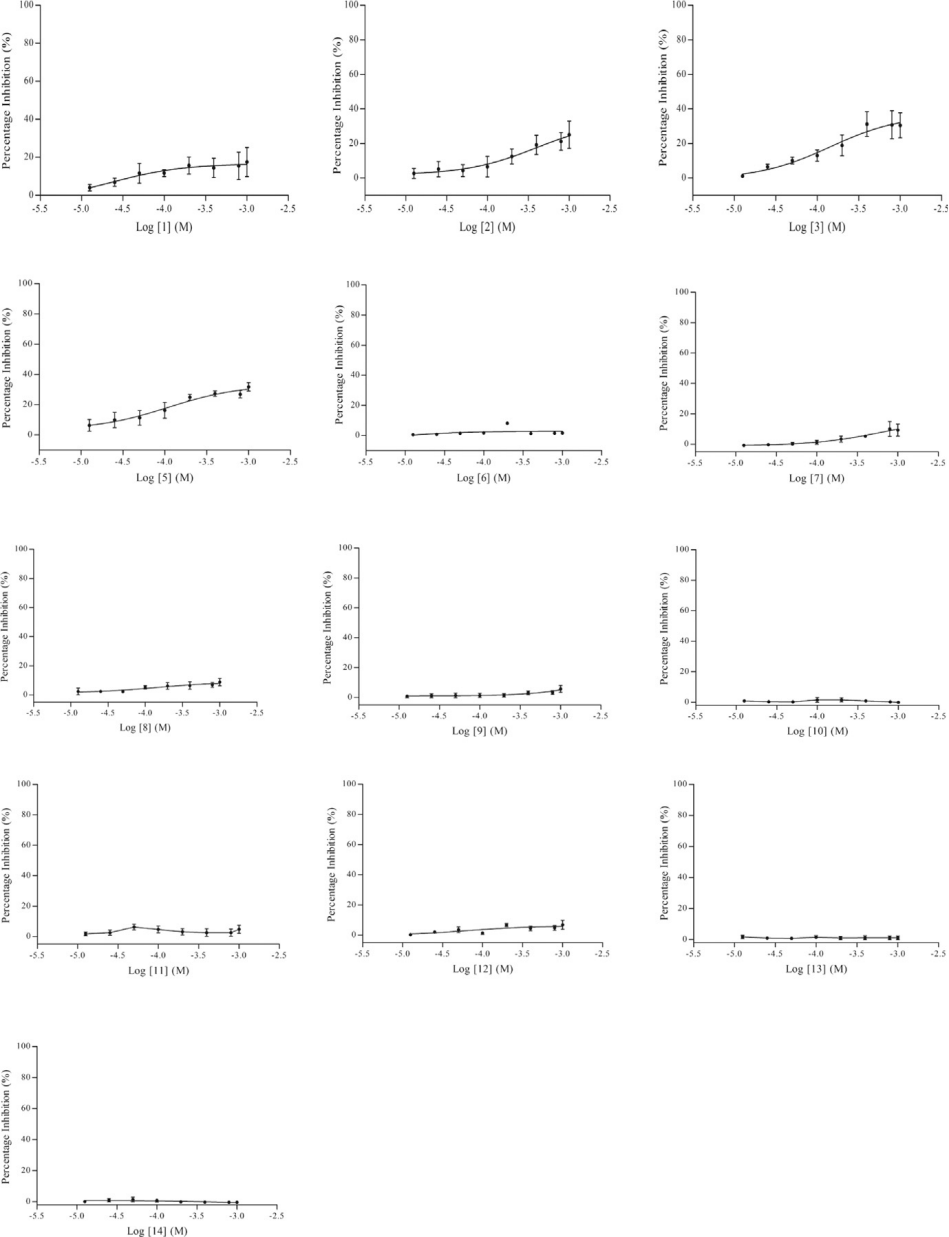
The effects of compounds on the NS2B/NS3 protease activity using AlphaScreen^®^ assay. Data are presented as mean alphasignal ± SEM of three independent experiments.

DMSO was used for preparing the stock solution of the compounds. Majority of the compounds were screened in the presence of 1% v/v DMSO, however, higher concentration of DMSO (10% v/v) was used for compounds with low solubility such as compound 8, 9, 10, 11 and 12. There were no significant differences in the alpha signal obtained between assays run in 1% v/v and up to 10% v/v DMSO. Using the AlphaScreen^®^ assay, aprotinin exhibited dose dependent inhibition against NS2B/NS3 enzyme with an IC_50_ value of 0.35 μM (Fig. 6a), which was comparable with the IC_50_ value generated by the protease assay (0.15 μM) (Fig. 6b). As for panduratin A, the maximum inhibition at the highest concentration tested (100 μM) in the AlphaScreen^®^ assay was just slightly above 50%, therefore, an IC_50_ value could not be determined within the concentration range used (Fig. 6c). On the other hand, the protease assay produced an IC_50_ value of 211.5 μM, which was expected (Fig. 6d). However, most of the derivatives were found to have no clear activity against NS2B/NS3 except compound 4 which demonstrated a weak activity. Both AlphaScreen^®^ and protease assay revealed a maximum inhibition of approximately 50% at the highest concentration tested (1 mM) (Fig. 6e and f). Higher concentrations of the both panduratin A and compound 4 were not tested using Alphascreen^®^ assay as the estimated IC_50_ values would be too high to qualify the compounds as potential NS2B/NS3 inhibitors. The poor activities of the rest of the thioguanine compounds are depicted in Fig. 7.

The interday variations were evaluated in assay plate replicates, with each plate containing wells of minimum and maximum signal controls. The mean value for the maximum signal groups on day 1, 2, 3 for aprotinin were 1.14 million cps (CV = 2.27%), 1.03 million cps (CV = 0.15%) and 1.30 million cps (CV = 3.49%), respectively. On the other hand, the mean value for the minimum signal was 5688 cps (CV = 3.90%), 6024 cps (CV = 5.41%), and 7256 cps (CV = 8.27%), respectively. The mean S/B ratio for aprotinin was 205:1. Similarly, the mean value for the maximum signal groups on day 1, 2, 3 for panduratin A were 1.67 million cps (CV = 3.15%), 1.27 million cps (CV = 2.61%) and 1.11 million cps (CV = 1.18%), respectively. Meanwhile, the mean value for the minimum signal was 6199 cps (CV = 6.22%), 3956 cps (CV = 4.22%), and 5191 cps (CV = 9.89%), respectively. The mean S/B ratio for panduratin A was 265:1. The interday variability of both maximum and minimum signals was generally less than 10%. Finally, the zʹ factor value for three independent experiments were calculated to determine the robustness and the quality of the developed assay. The individual zʹ factor of the optimized assay ranged between 0.36 to 0.65 for aprotinin, panduratin and compound 4, respectively, indicating that the assay are potentially useful for screening for anti-dengue activities [38, 41].

## 4 Discussion

Dengue infection is a global health problem and according to WHO, an approximately 40% of the world population are at risk of dengue infection [42]. The highest number of dengue cases that are mostly afflicted by DENV2 is reported from the South East Asian countries [43, 44]. Currently, small molecules drugs [6, 45], peptide drugs [46] and plant-derived medications or herbal medicines research for the treatment of dengue infection is gaining much interest [6, 21, 47, 48, 49]. However, the development of antiviral drugs to treat dengue infections remains a challenge, due to the limited *in vitro* high-throughput screening assays and suitable *in vivo* models to screen for anti-dengue drugs [50]. At present, no specific treatment, drugs and vaccine are available to protect human against dengue infection.

There are intensive efforts to develop drugs targeted against structural and/or nonstructural proteins involved in dengue viral replication, along with the development of vaccine. The first dengue vaccine, Dengvaxia^®^ by Sanofi Pasteur was registered in Mexico in December, 2015 but was reported to cause antibody dependent enhancement (ADE) and severe diseases in some cases [19, 20]. Meanwhile, other candidate vaccines such as PDK-53 vaccine [51] and rDEN4Δ30(ME) [52] were evaluated in clinical trials with unremarkable outcome. Development of peptide inhibitors targeting on NS2B/NS3 protease and DENV entry such as aldehyde peptide [53, 54], Bz-Nle-Lys-Arg-Arg-H [18], DET1-DET4 [55] and natural products were also explored for their potential antiviral activities [6, 21, 47]. For example, flavonoids were found to inhibit the NS2B/NS3 protease albeit weakly, with IC_50_ values ranging from 15 to 44 μM [49]. Other group of compounds studied for their anti-dengue activities include novel thiadiazoloacrylamide analogues with one of these showing moderate activity with IC_50_ values at 2.2 μM based on NS2B/NS3 protease assay [56]. Meanwhile, two nitro derivatives of 3,5-bis(arylidene)-4-piperidones were shown to inhibit NS2B/NS3 protease with IC_50_ values of 15.22 and 16.23 μM [57]. However, most of these antiviral compounds have only manifested moderate to weak anti-dengue activities. More efforts are certainly needed to discover novel inhibitors with significant anti-dengue activities.

AlphaScreen^®^ based assay is robust and has been widely utilized in various aspects of cell signaling research, drug discovery and screening platform and biomarker quantification [58]. In this study, the NS2B/NS3 target peptide substrate was designed and synthesized, whereby the substrate was tagged with a specific sequence and polyhistidine, respectively. This strategy is favorably applicable to any enzyme that possesses protease activity. Competitive displacement assays revealed that the peptide substrate was adequately tagged at both N and C-terminals. Subsequently, based on the cross-titration experiment and the typical bell-shaped curve, the optimum concentrations of substrate and protease were determined at 300 nM and 100 nM, respectively. As for the optimization, several assay parameters were evaluated prior to the screening of derivatives. Lower concentrations of HEPES appeared to produce higher signal intensity as compared to other types of buffer. Meanwhile, concentrations of NaCl do not appear to affect the signal much, therefore a lower concentration of NaCl (20 mM) was used in this assay. In previous study, the optimum activity of NS2B/NS3 protease was usually in the presence of low concentrations of NaCl [22, 59]. Quite surprisingly 0.03–0.3% (w/v) BSA in the presence of 10 mM HEPES and 20 mM NaCl improved the signal tremendously. Yang and co-workers have also used HEPES, NaCl and BSA for the assay solution in their AlphaScreen^®^ assay [41]. Highest maximum signal within the range of 1.4 million cps was obtained at pH 9.0, indicating improved sensitivity [59, 60]. This observation concurred with other studies which stated that the activity of NS2B/NS3 increased sharply from pH 7 to 9 [22, 59]. A 24 h incubation period produced significantly lower minimum signal which simply indicated that longer time was needed for the peptide substrate to be completely cleaved by the protease. This may be due to the reasonably low specific activity of NS2B/NS3 protease produced in the laboratory and improving the specific activity may reduce the incubation period. The final optimum assay solution in this assay consisted of 10 mM HEPES, 20 mM NaCl, 0.2% (w/v) BSA and at pH 9.

Subsequently known compounds such as aprotinin and a natural compound reported to have some NS2B/NS3 protease inhibitory activities such as panduratin A was evaluated using the optimized assay. Aprotinin, a broad spectrum protease inhibitor, was found to inhibit dengue NS2B/NS3 protease with an IC_50_ value of 0.35 μM using AlphaScreen^®^ assay, which was rather similar with the value derived using the protease assay. Interestingly, the published IC_50_ value of aprotinin was much lower (65 nM) in a different assay system on similar enzyme activity [59]. A difference in specific activity for the expressed proteases and type of substrates used may have contributed to this discrepancy. Surprisingly, panduratin A did not exhibit remarkable activity against NS2B/NS3 enzyme, as an IC_50_ value of more than 100 μM was expected based on the AlphaScreen^®^ assay. In fact, protease assay produced an IC_50_ value of 211.5 μM when higher concentration range of the compound was tested, a value higher than previously reported [57].

Appropriate and specific inhibitors that can be used as good controls in assays are currently lacking for the NS2B/NS3 enzyme. Studies by Leung and co-workers have found that even standard serine protease inhibitors such as 4-(2-aminoethyl) benzenesulfonyl fluoride.hydrochloride and N-Tosyl-L-phenylalanine chloromethyl ketone showed only 20–30% inhibition of this enzyme at 500 μM and 1 mM, respectively. Other serine protease inhibitors for which no enzyme inhibition was found included soybean trypsin inhibitor, 4-Amidinophenylmethanesulfonyl fluoride.hydrochloride, phenylmethylsulfonyl fluoride, leupeptin, pepstatin A, benzamidine and N-Tosyl-L-lysine chloromethyl ketone.hydrochloride [59]. On the other hand, aprotinin is not a specific NS2B/NS3 inhibitor, thus may be an added limitation for such assays. Both AlphaScreen^®^ and protease assays have indicated that panduratin A activity on NS2B/NS3 protease was weak and thus, may not be suitable to be used as positive control inhibitor for NS2B/NS3 protease. Tan and co-workers initially determined the NS2B/NS3 inhibitory activities of hydroxypanduratin and panduratin A using protease assay and found that concentration as high as 200 μM (or 80 ppm) was needed to inhibit 65-80% of the protease activity. K_i_ values derived were 21 and 25 μM, respectively, indicating moderate to weak inhibitory effects [21]. However, (8-HQ)-aminobenzothiazole derivatives with 2-aminothiazole or 2-aminobenzothiazole scaffold synthesized by Lai and co-workers, produced low IC_50_ values in the *in vitro* protease assays [61]. The most potent 8-HQ-aminobenzothiazole inhibitor has an IC_50_ value of 0.91 ± 0.05 μM and K_i_ value of 2.36 ± 0.13 μM [61]. This competitive inhibitor for DENV2 NS2B/NS3 protease may be useful as positive controls in assays targeting this protease.

Unfortunately, none of the fourteen derivatives showed significant inhibitory activity against dengue NS2B/NS3 protease despite earlier *in silico* simulation results suggested otherwise. Only compound 4 was found to weakly inhibit the NS2B/NS3 protease with an IC_50_ value probably near to 1 mM as determined using both AlphaScreen^®^ and protease assays. Although the calculated FEB usually correlates with the binding affinity and that the lower energy obtained, the stronger the binding affinity and the more likelihood to exhibit inhibitory activity against the protease, this was not observed in majority of the synthesized compounds. Currently no DENV NS2B/NS3 protease inhibitors have advanced to clinical trials despite numerous efforts to find potent inhibitors [15]. A main hurdle for such difficulty in designing small molecule inhibitors includes the nature of the NS2B/NS3 protease that has a shallow and hydrophilic catalytic site and that the active site preferentially binds substrates with basic (positively charged) residues [62]. For an inhibitor to bind, the substrate binding site requires a substantial conformational change of the NS2B fragment, thus, designing inhibitors by structure-based design has been challenging [63]. Hence, other strategies to identify inhibitors that bind to other region of the protein to inhibit its function would probably to overcome the hurdles.

Recently, a study has attempted to develop a conformational switch assay based on split luciferase complementation to monitor conformational change of NS2B and to identify candidate allosteric inhibitors [62]. In this assay, N- and C-terminal fragments of luciferase are genetically fused to a protein pair of interest, and would only emit light when there is interaction between two proteins. This mechanism was used to monitor the conformational changes of NS2B triggered upon binding of an active site inhibitor to the NS2B/NS3 protease complex and was used to identify and characterize allosteric inhibitors that prevent NS2B from forming the active conformation [62]. And interestingly a compound was found inhibit the conformational change of NS2B and significantly reduce titers of DENV2 with low EC_50_ values, although it was proved to be broad spectrum and not specific to NS2B/NS3 [62].

This is a first report on the utilization of AlphaScreen^®^ beads for screening of dengue NS2B/NS3 protease inhibitory activity. Although Takahashi and co-workers used similar method to examine the interaction between NS3 and NS5, these interactions could also serve as targets for the development of new antivirals against DENV [64]. As a conclusion, based on the zʹ factor, coefficient variance and S/B ratios, this AlphaScreen^®^ assay to screen for NS2B/NS3 protease inhibitors is potentially applicable for use in high throughput screening [38, 41]. Further optimization in the expression and purification of NS2B/NS3 protease are needed to obtain higher activity and using newer specific inhibitors may improve the reliability of the assay. Although a thioguanine derivative with a weak NS2B/NS3 protease inhibition activity was identified, this compound may be used as a starting compound for further modifications and/or improvements. More comprehensive molecular simulation is required.

## Declarations

### Author contribution statement

Muhammad Asyraf Abduraman, Maywan Hariono: Performed the experiments, Analyzed and interpreted the data, Wrote the paper.

Rohana Yusof, Noorsaadah Abd Rahman: Conceived and designed the experiments.

Habibah A. Wahab, Mei Lan Tan: Conceived and designed the experiments, Analyzed and interpreted the data, Wrote the paper.

### Funding statement

This work was supported by Sciencefund Grant from the Ministry of Energy, Science, Technology, Environment and Climate Change, Malaysia (MESTECC) (Grant No 02-05-23-SF0006), which was awarded to the corresponding author (Mei Lan Tan).

### Competing interest statement

The authors declare no conflict of interest.

### Additional information

Data associated with this study will be made available upon request to the corresponding authors.

## References

[1] Li G.H., Ning Z.J., Liu Y.M., Li X.H. (2017). Neurological manifestations of dengue infection. Front Cell Infect Microbiol.

[2] Rodenhuis-Zybert I.A., Wilschut J., Smit J. (2010). Dengue virus life cycle: viral and host factors modulating infectivity. Cell. Mol. Life Sci..

[3] Dhenni R., Karyanti M.R., Putri N.D., Yohan B., Yudhaputri F.A., Ma'roef C.N. (2018). Isolation and complete genome analysis of neurotropic dengue virus serotype 3 from the cerebrospinal fluid of an encephalitis patient. PLOS Negl Trop Dis.

[4] Guzman M.G., Alvarez M., Halstead S.B. (2013). Secondary infection as a risk factor for dengue hemorrhagic fever/dengue shock syndrome: an historical perspective and role of antibody-dependent enhancement of infection. Arch. Virol..

[5] Chung K.Y., Dong H., Chao A.T., Shi P.-Y., Lescar J., Lim S.P. (2010). Higher catalytic efficiency of N-7-methylation is responsible for processive N-7 and 2′-O methyltransferase activity in dengue virus. Virol.

[6] Frimayanti N., Chee C.F., Zain S.M., Rahman N.A. (2011). Design of new competitive dengue NS2B/NS3 protease inhibitors-a computational approach. Int. J. Mol. Sci..

[7] Tomlinson S.M., Watowich S.J. (2011). Anthracene-based inhibitors of dengue virus NS2B-NS3 protease. Antivir. Res..

[8] Preugschat F., Yao C.W., Strauss J.H. (1990). *In vitro* processing of dengue virus type 2 nonstructural proteins NS2A, NS2B, and NS3. J. Virol..

[9] Falgout B., Pethel M., Zhang Y.-M., Lai C.-J. (1991). Both nonstructural proteins NS2B and NS3 are required for the proteolytic processing of dengue virus nonstructural proteins. J. Virol..

[10] Chambers T.J., Hahn C.S., Galler R., Rice C.M. (1990). Flavivirus genome organization, expression, and replication. Annu. Rev. Microbiol..

[11] Markoff L. (1989). In vitro processing of dengue virus structural proteins: cleavage of the pre-membrane protein. J. Virol..

[12] Yang C.C., Hsieh Y.C., Lee S.J., Wu S.H., Liao C.L., Tsao C.H. (2011). Novel dengue virus-specific NS2B/NS3 protease inhibitor, BP2109, discovered by a high-throughput screening assay. Antimicrob. Agents Chemother..

[13] Tan W.L., Lee Y.K., Ho Y.F., Yusof R., Abdul Rahman N., Karsani S.A. (2018). Comparative proteomics reveals that YK51, a 4-Hydroxypandurantin-A analogue, downregulates the expression of proteins associated with dengue virus infection. PeerJ.

[14] Falgout B., Miller R.H., Lai C.-J. (1993). Deletion analysis of dengue virus type 4 nonstructural protein NS2B: identification of a domain required for NS2B-NS3 protease activity. J. Virol..

[15] Takagi Y., Matsui K., Nobori H., Maeda H., Sato A., Kurosu T. (2017). Discovery of novel cyclic peptide inhibitors of dengue virus NS2B-NS3 protease with antiviral activity. Bioorg. Med. Chem. Lett.

[16] Dodson G., Wlodawer A. (1998). Catalytic triads and their relatives. Trends Biochem. Sci..

[17] Wichapong K., N A., Pianwanit S., Sippl W., Kokpol S. (2013). Identification of potential hit compounds for dengue virus NS2B/NS3 protease inhibitors by combining virtual screening and binding free energy calculations. Trop. Biomed..

[18] Wichapong K., Pianwanit S., Sippl W., Kokpol S. (2010). Homology modeling and molecular dynamics simulations of dengue virus NS2B/NS3 protease: insight into molecular interaction. J. Mol. Recogn..

[19] Villar L., Dayan G.H., Arredondo-Garcia J.L., Rivera D.M., Cunha R., Deseda C. (2015). Efficacy of a tetravalent dengue vaccine in children in Latin America. N. Engl. J. Med..

[20] Capeding M.R., Tran N.H., Hadinegoro S.R., Ismail H.I., Chotpitayasunondh T., Chua M.N. (2014). Clinical efficacy and safety of a novel tetravalent dengue vaccine in healthy children in Asia: a phase 3, randomised, observer-masked, placebo-controlled trial. Lancet.

[21] Tan S.K., Pippen R., Yusof R., Ibrahim H., Khalid N., Rahman N.A. (2006). Inhibitory activity of cyclohexenyl chalcone derivatives and flavonoids of fingerroot, *Boesenbergia rotunda* (L.), towards dengue-2 virus NS3 protease. Bioorg. Med. Chem. Lett.

[22] Yusof R., Clum S., Wetzel M., Murthy H.M., Padmanabhan R. (2000). Purified NS2B/NS3 serine protease of dengue virus type 2 exhibits cofactor NS2B dependence for cleavage of substrates with dibasic amino acids in vitro. J. Biol. Chem..

[23] Shum D., Smith J.L., Hirsch A.J., Bhinder B., Radu C., Stein D.A. (2010). High-content assay to identify inhibitors of dengue virus infection. Assay Drug Dev. Technol..

[24] Liu L., Wen K., Li J., Hu D., Huang Y., Qiu L. (2012). Comparison of plaque- and Enzyme-Linked Immunospot-Based Assays to measure the neutralizing activities of monoclonal antibodies specific to domain III of dengue virus envelope protein. Clin. Vaccine Immunol..

[25] Timiryasova T.M., Bonaparte M.I., Luo P., Zedar R., Hu B.T., Hildreth S.W. (2013). Optimization and validation of a plaque reduction neutralization test for the detection of neutralizing antibodies to four serotypes of dengue virus used in support of dengue vaccine development. Am. J. Trop. Med. Hyg..

[26] Thomas S.J., Nisalak A., Anderson K.B., Libraty D.H., Kalayanarooj S., Vaughn D.W. (2009). Dengue plaque reduction neutralization test (PRNT) in primary and secondary dengue virus infections: how alterations in assay conditions impact performance. Am. J. Trop. Med. Hyg..

[27] Hsieh M.-S., Chen M.-Y., Hsieh C.-H., Pan C.-H., Yu G.-Y., Chen H.-W. (2017). Detection and quantification of dengue virus using a novel biosensor system based on dengue NS3 protease activity. PLoS One.

[28] Eglen R.M., Reisine T., Roby P., Rouleau N., Illy C., Bosse R. (2008). The use of AlphaScreen technology in HTS: current status. Curr. Chem. Genom..

[29] Prakash S., Kannapiran E., Ramasubburayan R., Lyapparaj P., Ananthi S., Palavesam A. (2011). Production and partial purification of protease by selected bacterial strains using raw milk as substrate. Malays. J. Microbiol..

[30] Hosseininaveh V., Bandani A., Hosseininaveh F. (2009). Digestive proteolytic activity in the Sunn pest, Eurygaster integriceps. J. Insect Sci..

[31] Schuttelkopf A.W., van Aalten D.M. (2004). PRODRG: a tool for high-throughput crystallography of protein-ligand complexes. Acta Crystallogr. D Biol. Crystallogr..

[32] Morris G.M., Huey R., Lindstrom W., Sanner M.F., Belew R.K., Goodsell D.S. (2009). AutoDock4 and AutoDockTools4: automated docking with selective receptor flexibility. J. Comput. Chem..

[33] Salvatore R.N., Smith R.A., NA K., Gavin T. (2005). A mild and highly convenient chemoselective alkylation of thiols using Cs2CO3-TBAI. Tetrahedron Lett..

[34] Panneerselvam P., Nair R.R., Vijayalakshmi G., Subramaniam E.H., Sridhar S.K. (2005). Synthesis of schiff bases of 4-(4-aminophenyl)-morpholine as potential antimicrobial agents. Eur. J. Med. Chem..

[35] Khumthong R., Angsuthanasombat C., Panyim S., Katzenmeier G. (2002). *In vitro* determination of dengue virus type 2 NS2B-NS3 protease activity with fluorescent peptide substrates. J. Biochem. Mol. Biol..

[36] Khumthong R., Niyomrattanakit P., Chanprapaph S., Angsuthanasombat C., Panyim S., Katzenmeier G. (2003). Steady-state cleavage kinetics for dengue virus type 2 NS2B-NS3(pro) serine protease with synthetic peptides. Protein Pept. Lett..

[37] Ayala J., Pimienta E., Rodriguez C., Anne J., Vallin C., Milanes M. (2013). Use of Strep-tag II for rapid detection and purification of *Mycobacterium tuberculosis* recombinant antigens secreted by *Streptomyces lividans*. J. Microbiol. Methods.

[38] Zhang J.H., Chung T.D.Y., Oldenburg K.R. (1999). A simple statistical parameter for use in evaluation and validation of high throughput screening assays. J. Biomol. Screen.

[39] Lee Y.K., Tan S.K., Wahab H.A., Yusof R., Rahman N.A. (2007). Nonsubstrate based inhibitors of dengue virus serine protease: a molecular docking approach to study binding interactions between protease and inhibitors. Asia Pac. J. Mol. Biol. Biotechnol..

[40] Brinkworth R.I., Fairlie D.P., Leung D., Young P.R. (1999). Homology model of the dengue 2 virus NS3 protease: putative interactions with both substrate and NS2B cofactor. J. Gen. Virol..

[41] Yang W., Wang L., Paschen W. (2013). Development of a high-throughput screening assay for inhibitors of small ubiquitin-like modifier proteases. J. Biomol. Screen.

[42] WHO (2014). World Health Day 2014. http://www.who.int/campaigns/world-health-day/2014/en/.

[43] Beaute J., Vong S. (2010). Cost and disease burden of dengue in Cambodia. BMC Public Health.

[44] Ahmad Nizal M., Rozita H., Mazrura S., Zainudin M., Hidayatulfathi O., Faridah M. (2012). Dengue infections and circulating serotypes in Negeri Sembilan, Malaysia. Malays. J. Public Health Med..

[45] Hariono M., Wahab H.A., Tan M.L., Rosli M.M., Razak I.A. (2014). 9-Benzyl-6-benzyl-sulfanyl-9H-purin-2-amine. Acta Crystallogr. Sect. E Struct. Rep. Online.

[46] Chew M.F., Poh K.S., Poh C.L. (2017). Peptides as therapeutic agents for dengue virus. Int. J. Med. Sci..

[47] Tan S.K., Pippen R., Yusof R., Rahman N.A., Ibrahim H., Khalid N. (2006). Screening of selected zingiberaceae extracts for dengue-2 virus protease inhibitory activities. Sunway Acad. J..

[48] Kadir S.L.A., Yaakob H., Zulkifli R.M. (2013). Potential anti-dengue medicinal plants: a review. J. Nat. Med..

[49] de Sousa L.R., Wu H., Nebo L., Fernandes J.B., da Silva M.F., Kiefer W. (2015). Flavonoids as noncompetitive inhibitors of Dengue virus NS2B-NS3 protease: inhibition kinetics and docking studies. Bioorg. Med. Chem..

[50] Noble C.G., Chen Y.L., Dong H., Gu F., Lim S.P., Schul W. (2010). Strategies for development of dengue virus inhibitors. Antivir. Res..

[51] Butrapet S., Huang C.Y., Pierro D.J., Bhamarapravati N., Gubler D.J., Kinney R.M. (2000). Attenuation markers of a candidate dengue type 2 vaccine virus, strain 16681 (PDK-53), are defined by mutations in the 5' noncoding region and nonstructural proteins 1 and 3. J. Virol..

[52] Blaney J.E., Sathe N.S., Hanson C.T., Firestone C.Y., Murphy B.R., Whitehead S.S. (2007). Vaccine candidates for dengue virus type 1 (DEN1) generated by replacement of the structural genes of rDEN4 and rDEN4Delta30 with those of DEN1. Virol. J..

[53] Yin Z., Patel S.J., Wang W.L., Chan W.L., Ranga Rao K.R., Wang G. (2006). Peptide inhibitors of dengue virus NS3 protease. Part 2: SAR study of tetrapeptide aldehyde inhibitors. Bioorg. Med. Chem. Lett.

[54] Yin Z., Patel S.J., Wang W.L., Wang G., Chan W.L., Rao K.R. (2006). Peptide inhibitors of Dengue virus NS3 protease. Part 1: Warhead. Bioorg. Med. Chem. Lett.

[55] Alhoot M.A., Rathinam A.K., Wang S.M., Manikam R., Sekaran S.D. (2013). Inhibition of dengue virus entry into target cells using synthetic antiviral peptides. Int. J. Med. Sci..

[56] Liu H., Wu R., Sun Y., Ye Y., Chen J., Luo X. (2014). Identification of novel thiadiazoloacrylamide analogues as inhibitors of dengue-2 virus NS2B/NS3 protease. Bioorg. Med. Chem..

[57] Osman H., Idris N.H., Kamarulzaman E.E., Wahab H.A., Hassan M.Z. (2017). 3,5-Bis(arylidene)-4-piperidones as potential dengue protease inhibitors. Acta Pharm. Sin. B.

[58] Yasgar A., Jadhav A., Simeonov A., Coussens N.P. (2016). AlphaScreen-based assays: ultra-high-throughput screening for small-molecule inhibitors of challenging enzymes and protein-protein interactions. Methods Mol. Biol..

[59] Leung D., Schroder K., White H., Fang N.X., Stoermer M.J., Abbenante G. (2001). Activity of recombinant dengue 2 virus NS3 protease in the presence of a truncated NS2B co-factor, small peptide substrates, and inhibitors. J. Biol. Chem..

[60] Wigle T.J., Herold J.M., Senisterra G.A., Vedadi M., Kireev D.B., Arrowsmith C.H. (2010). Screening for inhibitors of low-affinity epigenetic peptide-protein interactions: an AlphaScreen-based assay for antagonists of methyl-lysine binding proteins. J. Biomol. Screen.

[61] Lai H., Sridhar Prasad G., Padmanabhan R. (2013). Characterization of 8-hydroxyquinoline derivatives containing aminobenzothiazole as inhibitors of dengue virus type 2 protease in vitro. Antivir. Res..

[62] Brecher M., Li Z., Liu B., Zhang J., Koetzner C.A., Alifarag A. (2017). A conformational switch high-throughput screening assay and allosteric inhibition of the flavivirus NS2B-NS3 protease. PLoS Pathog..

[63] Aguilera-Pesantes D., Robayo L.E., Mendez P.E., Mollocana D., Marrero-Ponce Y., Torres F.J. (2017). Discovering key residues of dengue virus NS2b-NS3-protease: new binding sites for antiviral inhibitors design. Biochem. Biophys. Res. Commun..

[64] Takahashi H., Takahashi C., Moreland N.J., Chang Y.T., Sawasaki T., Ryo A. (2012). Establishment of a robust dengue virus NS3-NS5 binding assay for identification of protein-protein interaction inhibitors. Antivir. Res..

